# Microvesicles Derived from Human Umbilical Cord Wharton’s Jelly Mesenchymal Stem Cells Attenuate Bladder Tumor Cell Growth *In Vitro* and *In Vivo*

**DOI:** 10.1371/journal.pone.0061366

**Published:** 2013-04-12

**Authors:** Shuai Wu, Guan-Qun Ju, Tao Du, Ying-Jian Zhu, Guo-Hua Liu

**Affiliations:** 1 Department of Urology, Shanghai First People’s Hospital, School of Medicine, Shanghai Jiao Tong University, Shanghai, China; 2 Department of Urology, Qingdao Municipal Hospital, Qingdao, Shandong, China; University of Chicago, United States of America

## Abstract

Several studies suggest that mesenchymal stem cells (MSCs) possess antitumor properties; however, the exact mechanisms remain unclear. Recently, microvesicles (MVs) are considered as a novel avenue intercellular communication, which may be a mediator in MSCs-related antitumor effect. In the present study, we evaluated whether MVs derived from human umbilical cord Wharton’s jelly mesenchymal stem cells (hWJMSCs) may inhibit bladder tumor T24 cells growth using cell culture and the BALB/c nu/nu mice xenograft model. CCK-8 assay and Ki-67 immunostaining were performed to estimate cell proliferation in vitro and in vivo. Flow cytometry and TUNEL assay were used to assess cell cycle and apoptosis. To study the conceivable mechanism by which hWJMSC-MVs attenuate bladder tumor T24 cells, we estimated the expression of Akt/p-Akt, p-p53, p21 and cleaved Caspase 3 by Western blot technique after exposing T24 cells to hWJMSC-MVs for 24, 48 and 72h. Our data indicated that hWJMSC-MVs can inhibit T24 cells proliferative viability via cell cycle arrest and induce apoptosis in T24 cells in vitro and in vivo. This study showed that hWJMSC-MVs down-regulated phosphorylation of Akt protein kinase and up-regulated cleaved Caspase 3 during the process of anti-proliferation and pro-apoptosis in T24 cells. These results demonstrate that hWJMSC-MVs play a vital role in hWJMSC-induced antitumor effect and may be a novel tool for cancer therapy as a new mechanism of cell-to-cell communication.

## Introduction

Recent studies indicate that multiple MSCs display anticancer activities on some specific cell lines in vitro and in vivo. Human bone marrow mesenchymal stem cells (hBMMSCs) given by tail vein injection possessed intrinsic tumor-suppressive properties in an in vivo mouse model of Kaposi’s sarcoma [1]. hBMMSCs’ inhibitory effect against Non-Hodgkin’s lymphoma cells in SCID mice also was reported by Secchiero and his colleagues [2]. Both umbilical cord stem cells originated from human and rat could abolish the breast cancer cells according to Ayuzawa [3] and Ganta’s [4] studies. Several studies reported results about the effects made by MSCs’ immunosuppressive action [5], trans-differentiation [6], [7] or acting on tumor cells by various factors secreted from MSCs [8]. Recently, more and more scientific researchers are focusing on MVs which are released from multiple cell types, including mesenchymal stem cells [9]–[13], into tumor microenvironment. MVs may play a pivotal role as mediators of extracellular communication in the development and growth of human malignancies [14]–[17]. MVs are heterogeneous in size ranging from 30 to 1,000 nm in diameter [18]–[20], and exhibit pleiotropic biological function as a novel avenue for cell-to-cell communication. MVs may influence the behavior of the recipient cells in different ways: a) directly stimulate the cells by a surface interaction [21]; b) transfer receptors from the cell of origin to the target cell [22]; c) deliver proteins to target cells [23], [24]; d) mediate a horizontal transfer of mRNA and microRNA inducing epigenetic changes in the target cell [10], [25]–[27]. Therefore, understanding the modulation of MVs inhibitory effect upon tumor cells may provide insight into the molecular mechanisms that underlie MSCs antitumor effect.

In the present study, we attempted to evaluate whether hWJMSC-MVs may attenuate the growth of bladder tumor T24 cells in vitro and in vivo. We treated T24 cells with diverse concentrations hWJMSC-MVs and then analyzed the T24 cells with CCK-8 assay, flow cytometry to estimate cell viability, cell cycle and apoptosis. We also studied the expression of Akt/p-Akt, p-p53, p21, cleaved Caspase 3 with Western-blotting methods. In vivo, we subcutaneously transplanted T24 cells combining with or without hWJMSC-MVs into nude mice and measured the tumor size to estimate the inhibition of hWJMSC-MVs on T24 cells. T24 tumor tissues were further analyzed with H&E staining, immunohistochemistry staining and TUNEL assay (MATERIALS AND METHODS). Our data showed that hWJMSC-MVs can be extracted successfully from the supernatant of hWJMSCs culture media and observed with transmission electron microscopy ranging from 30 to 500 nm in diameter (RESULTS). Notably, we found hWJMSC-MVs exert anti-proliferative and a pro-apoptotic effect on T24 cells both in vitro and in vivo which appear to be mediated by potently down-regulating phosphorylation of Akt protein kinase and activating p53/p21 and Caspase 3 (RESULTS OR CONCLUSION SECTION).

## Materials and Methods

### Ethics statement

In this study, all research involving human participants was approved by the institutional review board of the Chinese Academy of Medical Science and Medical School of Shanghai Jiao Tong University. Human individuals in this study gave written informed consent to participate in research and allow us to publish the case details. This study was carried out in strict accordance with the recommendations in the Guide for the Care and Use of Laboratory Animals of Shanghai Jiao Tong University. The protocol was approved by the Committee on the Ethics of Animal Experiments of Shanghai Jiao Tong University. All surgery was performed under sodium pentobarbital anesthesia, and all efforts were made to minimize suffering.

### Cell culture

Human umbilical cords were aseptically collected from full-term cesarean-section infants at Shanghai Jiao Tong University, affiliated to the First People’s Hospital. Umbilical cords were stored aseptically in cold Dulbecco's modified Eagle medium (DMEM) and then cellular isolation started within 4h from partum. The hWJMSCs isolation was performed as described previously [28], [29]. Briefly, the umbilical cord was pulverized into approximately 1–2 mm^3^ pieces after the blood cells were removed away from the arteries and vein. The cord pieces were transferred to a Petri dish containing DMEM with low glucose (DMED-LG, Gibco) supplemented with 10% fetal bovine serum (FBS, Gibco) at 37°C in a humidified atmosphere with 5% CO_2_. The medium was changed every two days. After two weeks, the adherent cells were harvested with 0.25% trypsin (Gibco) treatment and subcultured. Only cells from passage 3 to 6 were used for experiments.

Bladder transitional cell carcinoma lines (T24) and human foreskin fibroblast (HFF) were obtained from a commercial source (Shanghai Institutes for Biological Sciences, Shanghai, China) and cultured in RPMI-1640 (Gibco) supplemented with 10% FBS.

### Isolation of MVs

MVs were obtained from supernatants of hWJMSCs and of HFFs as previously described [30], [31]. In brief, hWJMSCs were cultured in DMEM deprived of FBS and supplemented with 0.5% bovine serum albumin (BSA) (Sigma-Aldrich, St. Louis, MO) overnight. HFFs were cultured in serum-free RPMI-1640 supplemented with 0.5% BSA. The viability of both cell types cultured in respective medium overnight was > 99% for hWJMSCs and 90% for HFFs as detected by trypan blue exclusion. No apoptotic cells were detected by TUNEL assay in hWJMSCs and < 3% apoptotic cells were detected for HFFs. After centrifugation at 2,000 g for 20 minutes to remove debris, cell-free supernatants were ultracentrifuged at 100,000 g in a SW41 swing rotor (Beckman Coulter Optima L-80K ultracentrifuge; Beckman Coulter, Fullerton, CA) for 1h at 4°C. MVs were washed once with serum-free M199 (Sigma-Aldrich) containing 25 mM HEPES (PH 7.4) and submitted to a second ultracentrifugation in the same conditions. To quantify the protein content, MV pellets were suspended in serum-free M199 and estimated by a Bradford assay (Bradford protein assay kit, P0006, beyotime institute of biotechnology). Endotoxin contaminations of MVs were excluded by Limulus test according to the manufacturer’s instruction (Charles River Laboratories, Inc., Wilmington, MA, USA) and MVs were stored at –80°C.

### Transmission electron microscopy

MVs were fixed with 2.5% glutaraldehyde in PBS for 2h. After MVs were washed, they were ultracentrifuged and suspended in 100 µl PBS. A 20 µl drop of MVs was loaded onto a formvar/carbon-coated grid, negatively stained with 3% aqueous phosphotungstic acid for 1 minute and observed by transmission electron microscopy (HITACHI, H-7650, Japan).

### MVs surface marker analysis

Flow cytometry was used to characterize the isolated MVs. MVs were incubated for 30 minutes at room temperature, 5 µl latex beads (Aldehyde/sulphate latex 4% w/v 4 µm, Molecular Probes, Leiden, The Netherlands) were added and incubated for another 30 minutes at 4°C, then washed in 0.5% BSA in PBS and incubated with different antibodies (CD9, CD34, CD44, CD45, CD63, CD73, eBioscience, cat#12-0098-41, cat#11-0349-41, cat#11-0441-81, cat#11-0459-71, cat#25-0639-41, cat#11-0739-42, respectively) or with appropriate isotype controls IgG. After washing, MVs-coated beads were immediately analyzed using a FACS Calibur flow cytometer (BECTON DICKINSON FACSCalibur).

### Cell viability assay

The cell viability rate of T24 was evaluated using cell counting kit-8 (CCK-8 Kit, C0037, beyotime institute of biotechnology). Cells were seeded in 96-well plates (2500 cells/well) and incubated in fresh medium at 37°C in a 5% CO_2_ atmosphere for 24h. The cells were then washed with PBS, and the medium was replaced with fresh medium containing hWJMSC-MVs (100 or 200 µg/ml protein) or HFF-MVs (200 µg/ml protein). After 24, 48 and 72h, cells were washed with PBS and incubated in 100 µl RPMI-1640 containing 10 µl CCK-8 solution for another 3h. The absorbance of each well at 450 nm was spectrophotometrically measured using a microplate ELISA reader (model FL 311, Bio-Tek Instruments, Winooski, VT, USA). T24 cells cultured in RPMI-1640 without MVs and culture media without cells were used as negative and blank controls, respectively. Following the deduction of the blank cell absorbance, the treated cell proliferation inhibition rate was expressed as a percentage of the absorbance decrease to control cell absorbance.

### Cell cycle analysis

For cell cycle analyses, T24 cells were cultured in the presence of hWJMSC-MVs (0, 50, 100, 200 µg/ml protein) for 48h. Cells were collected by trypsin treatment and fixed with 70% ice-cold ethanol overnight. The fixed cells were stained with 50 µg/ml propidium iodide (PI, Sigma) in PBS containing 0.1% Triton X-100 (Sigma) and 50 µg/ml RNase (Sigma), and then analyzed using a FACS Calibur flow cytometer (BECTON DICKINSON FACSCalibur). The experiments were repeated three times to ensure reproducibility.

### Annexin V/PI assay

To analyze the effect of hWJMSC-MVs against T24 cells, apoptosis assays were performed using Annexin V/PI staining. T24 cells were cultured in 6-well plates (4×10^4^ cells/well) in RPMI-1640 containing hWJMSC-MVs (100 or 200 µg/ml protein) for 24, 48 and 72h. At the end of the incubation times, cells were collected, washed once with PBS, and then washed with Annexin V binding buffer (1×). The cells were stained with 3.5 µl Annexin V-fluorescein isothiocyanate (FITC) at room temperature for 10 minutes and then counterstained with 3.5 µl PI and finally analyzed using a FACS Calibur flow cytometer (BECTON DICKINSON FACSCalibur). T24 cells cultured in fresh RPMI-1640 without hWJMSC-MVs were used as control. All Annexin V positive cells were considered to have apoptosis. FlowJo software version 7.6.1 (Tree Star, CA, USA) was used for data analysis.

### Western blotting

T24 cells were seeded in 6-well plates in fresh RPMI-1640 media for 12h initial culture, and then changed with RPMI-1640 containing hWJMSC-MVs (0 or 200 µg/ml protein) for another 24, 48 and 72h. T24 cells were then washed with PBS three times on ice and lysed with 100 µl lysis buffer supplemented with protease and phosphatase inhibitors. Cell lysates were collected by scraping and then centrifuged at 10,303 g for 15 minutes. Protein concentration was measured with BCA Protein Assay, and adjusted to equivalent amounts. 20–30 µg of total protein from each sample were electrophoresed on an 8–10% SDS-PAGE gel and transferred onto nitrocellulose membranes (Millipore). Membranes were blocked in 5% non-fat milk in TBS containing 0.1% Tween 20 (TBST) for 1h at room temperature, and then incubated with primary antibodies (Akt, p-Akt, Cleaved Caspase 3, p21, p-p53, GAPDH, β-Actin, Cell Signaling Technology, #5373, #5056, #8831, #9281, #9654, #5014, #4967, respectively) for 1–2h at room temperature. After washing and incubating with a HRP-conjugated secondary antibody, detected by ECL regent (Millipore Immobilon, Cat: WBKLS0500).

### Xenograft tumor model

To evaluate the in vivo effect of hWJMSC-MVs on T24 bladder tumor cells, twenty four 4-week-old male BALB/c nu/nu mice (Laboratory Animal Center of Shanghai, Academy of Science, China) were used. Mice were allowed to acclimate for 1 week after arrival. The twenty four mice were randomly divided into 4 groups (n = 6). All groups received subcutaneous injection of 200 µl PBS per mouse containing: (1) 1×10^7^ T24 cells; (2) 1×10^7^ T24 cells mixed with 1×10^7^ hWJMSCs; (3) 1×10^7^ T24 cells mixed with 200 µg protein hWJMSC-MVs; (4) 200 µg protein hWJMSC-MVs. Animals were monitored daily for changes in weight, tumor size, side effects of treatment and signs of any sickness. All the mice were sacrificed 30 days after tumor inoculation by cervical dislocation under sodium pentobarbital anesthesia. Tumor growth was determined by caliper measurement of the length and width of the tumor mass. Tumor volumes were calculated by the modified ellipsoidal formula: V = 1/2(length×width^2^) [32].

### Histopathology and immunohistochemistry

Animal specimens were fixed in 10% buffered-formalin solution and embedded in paraffin. For morphological analysis, hematoxylin-eosin (H&E) staining and microscopic examination were performed on 3-µm-thick sections from paraffin-embedded tumor blocks. To confirm that the tumor tissues were originated from human bladder tumor T24 cells, immunohistochemistry was performed using a polyclonal rabbit antibody against human nuclear mitotic apparatus protein (NuMA) according to the manufacturer’s instructions. Briefly, paraffin-embedded sections were labeled with NuMA (1:100, Abcam, Cambridge, UK), followed by a goat anti-rabbit secondary antibody using 3, 30 diaminobenzidine (DAB) reagents as substrate. Finally, Harris hematoxylin counterstaining was performed. In the same way, 3-µm-thick sections were stained using proliferation marker rabbit monoclonal to Ki-67 (1∶100, Abcam, Cambridge, UK) in order to assess the tumor cells proliferation (the Ki-67 labeling index) [33]. Data was analyzed by Image-pro Plus v6.0 software.

### TUNEL assay

For apoptosis quantification, T24 xenograft sections were processed for in situ immunocytochemical localization of nuclei exhibiting DNA fragmentation by the technique of terminal deoxynucleotidyl transferase (TdT)-mediated dUTP digoxigenin nick-end labeling (TUNEL) with the use of an apoptosis detection kit (In Situ Cell Death Detection Kit, POD, Roche, 11684817910). The protocols were followed according to the manufacturer’s instructions.

Paraffin-embedded samples (3-µm-thick sections) were deparaffinized and rehydrated with xylene and ethanol and permeabilized with 20 µg/ml of proteinase K (Gibco, Grand Island, NY). Endogenous peroxidase was inactivated by coating the samples with 3% H_2_O_2_. Sections were rinsed with PBS and then immersed 60 minutes in TdT buffer at 37°C and then incubated 30 minutes with the antidigoxigenin peroxidase conjugate, followed by the peroxidase substrate (3′-diaminobenzidine tetrahydrochloride [DAB]). Finally, sections were counterstained with 0.5% (wt/vol) methyl green. The apoptosis index was evaluated using a light microscope. Results were expressed as a percentage of TUNEL–positive to the total.

### Statistical analysis

Results from at least three independent experiments are reported as the means±standard deviation (SD). Statistical analyses were performed using SPSS v19.0. Statistical significance was assessed by 2-tailed Student’s t test for 2 groups and one-way analysis of variance (ANOVA) for more than 2 groups. A value of P<0.05 was considered to be statistically significant.

## Results

### Isolation and expansion of hWJMSCs

hWJMSCs presented predominantly spindle-shape morphology, a characteristic of monolayer growth pattern. Flow cytometry analysis showed the cells exhibit surface cell marker CD44, CD73, CD90, CD105, and negative for CD14, CD31, CD34, CD 45. (Data not show)

### Extraction and characterization of hWJMSC-MVs

**Figure 1:**
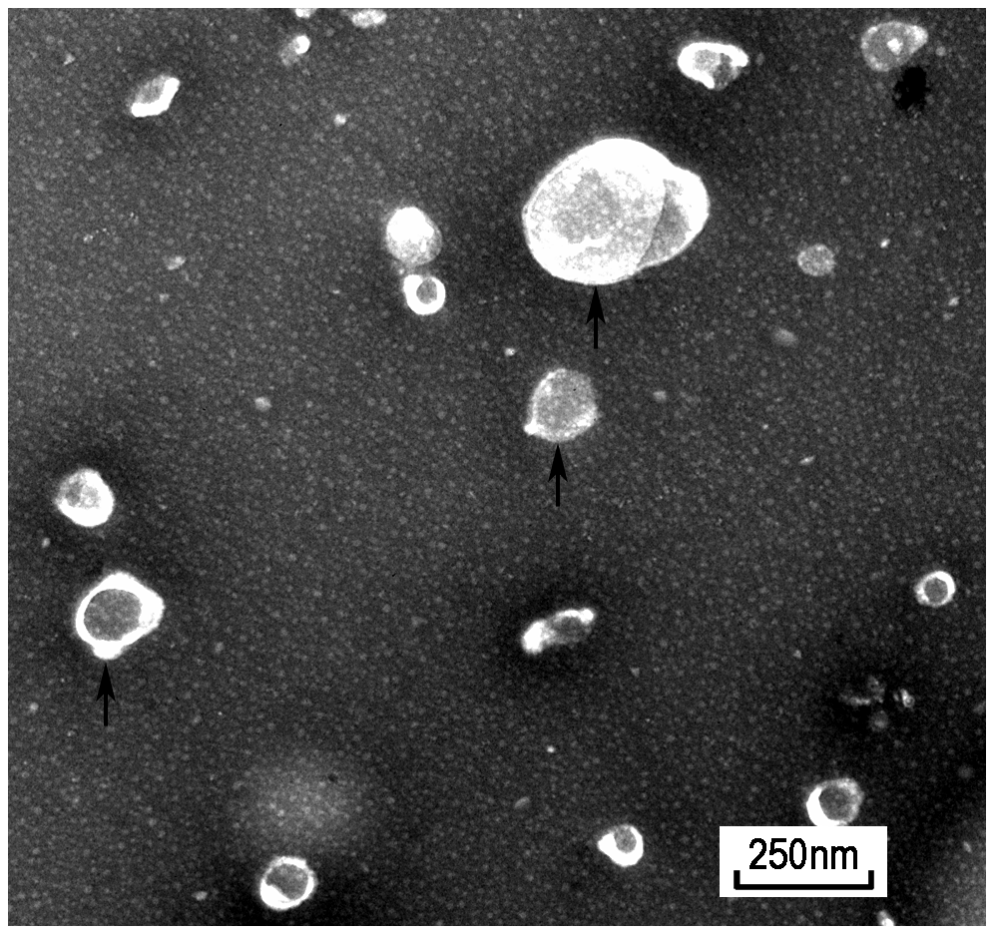
hWJMSC-MVs observed by transmission electron microscopy. hWJMSC-MVs were heterogeneous lipid bi-layer vesicles that range from 30–500 nm in diameter, and characterized by cup-shaped morphology. (black arrow) Scale bar  =  250 nm.

**Figure 2:**
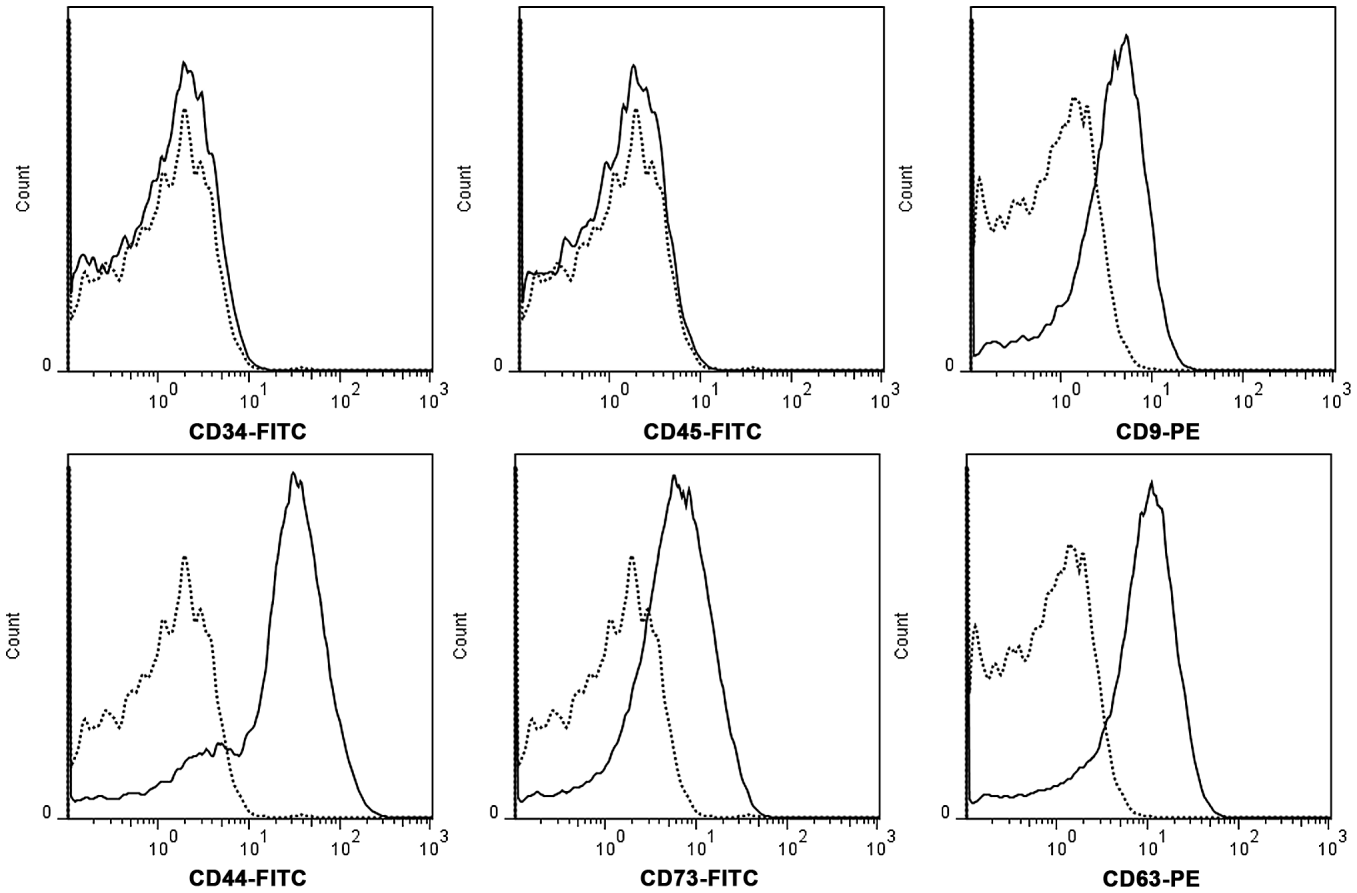
hWJMSC-MVs surface expressed molecules analysis. Flow cytometery analysis showed hWJMSC-MVs were positive for some surface expressed molecules typically expressed by MSCs, such as CD9, CD44, CD63, CD73, and negative for CD34, CD45.

hWJMSC-MVs were successfully isolated using ultracentrifugation. They were heterogeneous lipid bi-layer vesicles of approximately 30–500 nm in diameter, and characterized as cup-shaped or irregular-shaped (**Figure** **1**). Some bigger vesicle morphology changed dramatically as a result of the mode of electron microscopic tissue preparation as observed by electron transmission microscopy. Flow cytometry analysis showed hWJMSC-MVs were positive for some surface expressed molecules typically expressed by hWJMSCs, such as CD9, CD44, CD63, CD73, and negative for CD34, CD45 (**Figure 2**).

### Cell morphology

**Figure 3:**
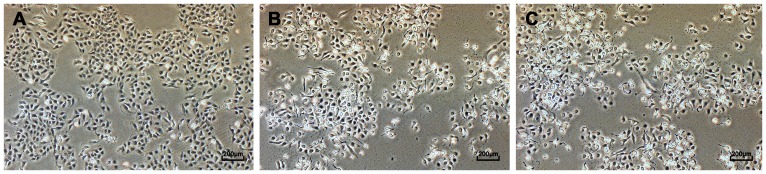
Cell morphology observed in light microscopy. T24 cells were observed after cultured in RPMI-1640 containing hWJMSC-MVs (0, 100 or 200 µg/ml protein) for 72h. Cells exposed to hWJMSC-MVs (B, 100 µg/ml protein; C, 200 µg/ml protein) for 72h showed decreases in cell number and production of degenerating/dead cells compared to control (A). They exhibited fragmentation, cell shrinkage, membrane damage, blebbing and cell debris. Scale bar  =  200 µm.

T24 cells maintained cobblestone-shape morphology, and grew well in fresh RPMI-1640 medium forming a confluent monolayer in culture. However, after changed to RPMI-1640 containing 100 or 200 µg/ml protein hWJMSC-MVs (**Figure 3B, C**) for 72h all T24 cells showed a decrease in cell number and production of degenerating/dead cells compared to control (**Figure 3A**). They exhibited fragmentation, cell shrinkage, membrane damage, blebbing and cell debris. Cells exposed to 200 µg/ml protein hWJMSC-MVs showed more changes in cell number and morphology than cells treated with 100 µg/ml protein hWJMSC-MVs.

### hWJMSC-MVs inhibit bladder tumor cells proliferation in vitro

**Figure 4:**
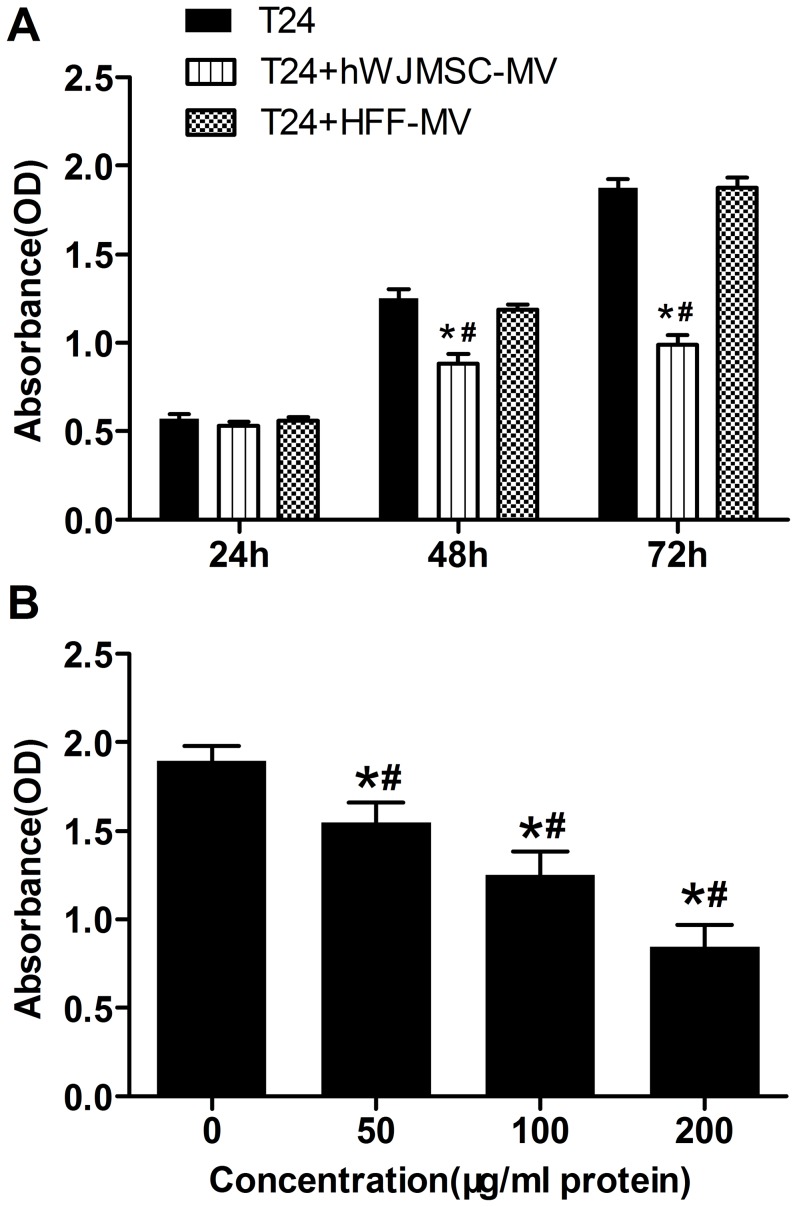
Cell proliferation assay (CCK-8 assay). A, T24 cells were analyzed with CCK-8 assay after cultured in RPMI-1640 containing hWJMSC-MVs (0 or 200 µg/ml protein) for 24, 48 and 72h. HFF-MVs were used as negative control. B, T24 cells were exposed to 0, 50, 100 or 200 µg/ml protein hWJMSC-MVs for 72h. The “*” and “#” indicate statistical significance at P<0.05 compared to respective controls within the group and between the groups, respectively.

To determine effect of hWJMSC-MVs on bladder tumor T24 cell growth in vitro, we evaluated the viability and progression of T24 cells exposed to hWJMSC-MVs (100 or 200 µg/ml protein) for 24, 48 and 72h. The CCK-8 assay showed that all T24 cells treated with hWJMSC-MVs (200 µg/ml protein) exhibited growth retardation both for 48 and 72h, and inhibited by 29.23% (±1.70) and 45.70% (±1.42) respectively, compared to controls (**Figure 4A**). However, there was no significant difference in cell proliferation activity between treated cells and control groups for 24h exposure. As a negative control, HFF-MVs have no significant inhibitory effect on T24 cell proliferation. T24 cells exposed to hWJMSC-MVs (50, 100, 200 µg/ml protein) exhibited signs of dose-dependent inhibition of cell proliferation at 72h (**Figure 4B**). The cells were significantly inhibited by 17.18% (±0.92), 33.87% (±3.28), 48.39% (±6.00) for the three concentrations of hWJMSC-MVs, respectively, compared to control. There was a significant difference between the groups (50, 100 and 200 µg/ml protein hWJMSC-MVs ) on the inhibition on T24 cells.

### hWJMSC-MVs arrest tumor cell cycle

**Figure 5:**
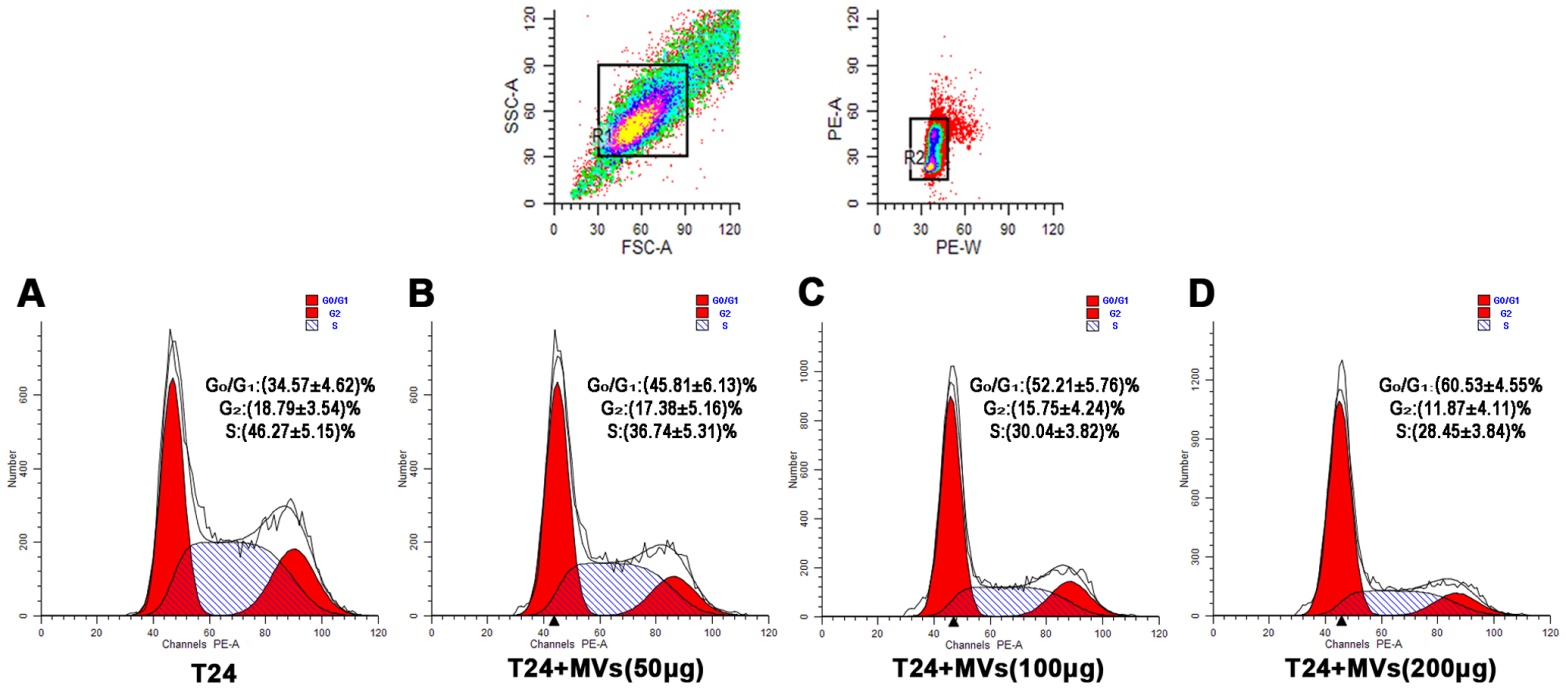
Cell cycle analysis. Cell cycle analyses were performed after T24 cells were treated with hWJMSC-MVs (0, 50, 100 and 200 µg/ml protein) for 48h. Compared to T24 cells alone (A), hWJMSC-MVs induced a significant increase of T24 cells in G_0_/G_1_ phase and a dramatic decrease in S phase (B, C, D, p<0.01). After being exposed to 200 µg/ml protein hWJMSC-MVs, the fraction of T24 cells in G_2_ phase was greatly decreased (D, p<0.01).

After T24 cells were incubated with various concentrations of hWJMSC-MVs, cell cycle analysis was evaluated (**Figure 5**). We observed a significant accumulation of T24 cells predominantly in G_0_/G_1_ phase ((34.57±4.62)%, (45.81±6.13)%, (52.21±5.76)%, (60.53±4.55)% for 0, 50, 100, 200 µg/ml protein hWJMSC-MVs, respectively) and a dramatic decrease in the S phase ((46.27±5.15)%, (36.74±5.31)%, (30.4±3.82)%, (28.45±3.84)% for 0, 50, 100, 200 µg/ml protein hWJMSC-MVs, respectively) in a dose-dependent manner. Compared to T24 cells alone, 200 µg/ml protein hWJMSC-MVs induced a significant decrease in percentage of cells in G_2_ phase ((18.79±3.54) % versus (11.87±4.11) %, p<0.01).

### hWJMSC-MVs promote bladder tumor cell apoptosis in vitro

**Figure 6:**
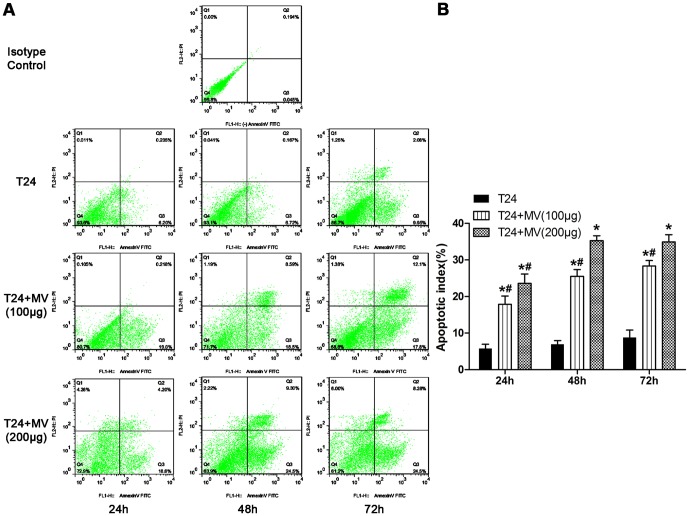
Apoptosis assay (Annexin V/PI assay). A, Apoptosis assay was performed after T24 cells were cultured in RPMI-1640 containing hWJMSC-MVs (0, 100 or 200 µg/ml protein) for 24, 48 and 72h, respectively. B, Apoptosis index analysis. Annexin V-FITC positive cells were expressed as mean±SD from three different replicates. The “*” and “#” indicate statistical significance at P<0.05 compared to respective controls within the group and between the groups, respectively.

To estimate the bladder tumor T24 cells apoptosis after treated with hWJMSC-MVs, an apoptosis assay was performed. Our data found that T24 cells increased in Annexin V-FITC positive cells after exposed to hWJMSC-MVs (100 or 200 µg/ml protein) for 24, 48 and 72h, compared to their respective controls. The apoptosis indices of the two groups (T24 cells +100 µg/ml protein hWJMSC-MVs, T24 +200 µg/ml protein hWJMSC-MVs) were 208.25% (±7.30), 265.23% (±7.46), 226.99% (±5.09) and 306.93% (±8.30), 405.51% (±8.84), 285.56% (±7.00) for 24, 48 and 72h, respectively, compared to their respective controls (**Figure 6A**). And all the increases were statistically significant except when exposed to 200 µg/ml protein hWJMSC-MVs for 48h versus 72h (**Figure 6B**). Notably, after T24 cells exposed to hWJMSC-MVs for 72h, there were more cell debris and naked cell nuclei as a result of cell death.

### Western blot

**Figure 7:**
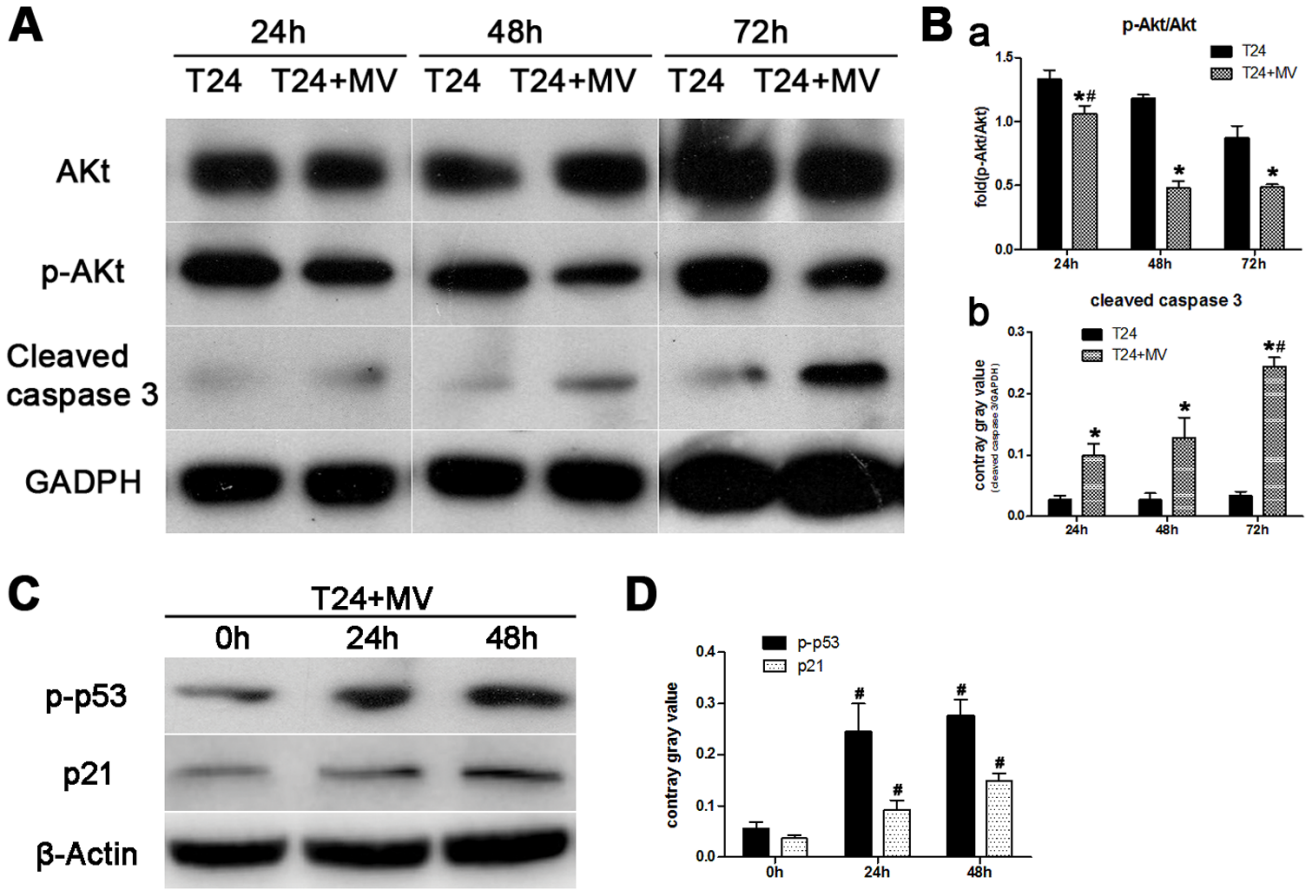
hWJMSC-MVs inhibited the phosphorylation of Akt and activated Caspase 3, p53 and p21. T24 cells were incubated with hWJMSC-MVs for indicated time periods, Akt, p-Akt, cleaved Caspase 3 expression were examined by Western blot analysis. A, After being exposed to hWJMSC-MVs for 24, 48 and 72h, T24 cell growth was significantly hindered by down-regulating the phosphorylation of Akt and up-regulating cleaved Caspase 3. B, Compared with T24 cells exposed to hWJMSC-MVs for only 24h, the increase of exposure time (48 or 72h) showed more effective inhibition on phosphorylation of Akt (p<0.01) (a). Conversely, expression of cleaved Caspase 3 was significantly up-regulated when T24 cells were exposed to hWJMSC-MVs for 72h than for 24 or 48h (p<0.01) (b). C, D, After being treated with hWJMSC-MVs for 24 and 48h, p-p53 and p21 were dramatically up-regulated in comparison with the controls (0h). Independent experiments were repeated up to three times with similar results. The “*” and “#” indicate statistical significance at P<0.05 in comparison with respective controls within the group and between the groups, respectively.

To identify the mechanism by which T24 cell growth is attenuated by hWJMSC-MVs, a Western blot analysis of proliferation- and apoptosis-related signal protein (Akt/p-Akt, cleaved Caspase 3, p-p53, p21) was performed. Our data found that hWJMSC-MVs significantly down-regulated T24 cell phosphorylation of Akt and activated Caspase 3 leading to low expression of p-Akt and high expression of cleaved Caspase 3 in comparison with respective controls after exposed to hWJMSC-MVs for 24, 48 and 72h (**Figure 7A, B**). However, there was no significant difference of total Akt expression between the groups. The increase of exposure time showed more effective inhibition on phosphorylation of Akt (24h versus (48 or 72h), p<0.01) (**Figure 7B** **, a**). Moreover, expression of cleaved Caspase 3 was significantly up-regulated when T24 cells exposed to hWJMSC-MVs for 72h than for 24 or 48h (p<0.01) (**Figure 7B** **, b**). Our data also found p-p53 and p21 were dramatically up-regulated in response to hWJMSC-MVs treatment (**Figure 7C, D**).

### hWJMSC-MVs significantly attenuate bladder tumor cell growth in vivo

**Figure 8:**
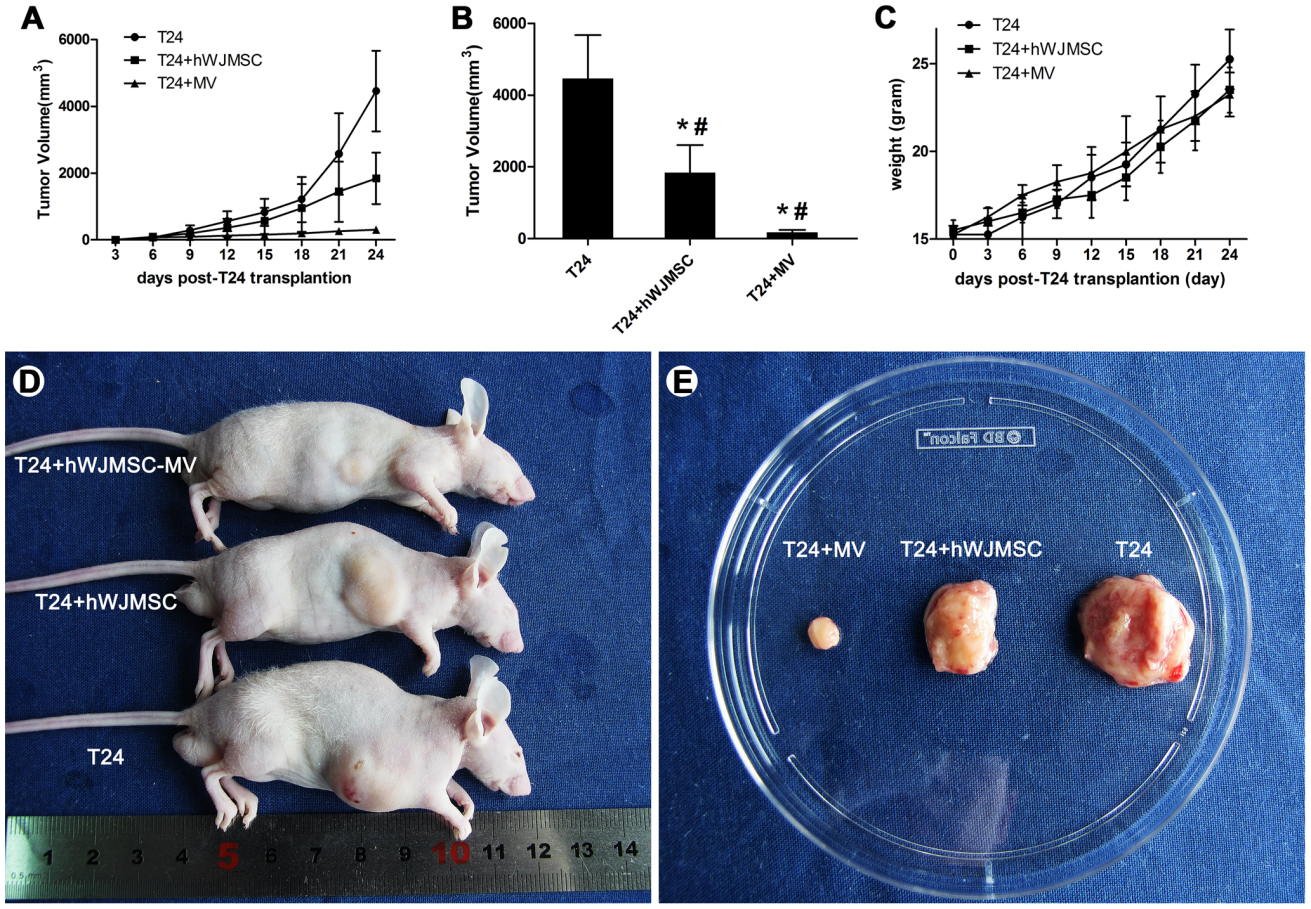
hWJMSC-MVs attenuated T24 xenograft growth in vivo. A, T24 tumor growth with time. B, the three groups (T24 cells alone, T24+hWJMSCs, T24+hWJMSC-MVs) tumor volume. C, the mice weight with time. D, representative examples of mice after transplantation with T24 cells alone, T24+hWJMSCs, or T24+hWJMSC-MVs subcutaneously for 28 days. E, Gross observation, hWJMSC-MVs, as well as hWJMSCs, significantly retarded T24 xenografts growth. The “*” and “#” indicate statistical significance at P<0.05 in comparison with respective controls within the group and between the groups, respectively.

We detected the ability of hWJMSC-MVs inhibition against bladder tumor cell T24 after subcutaneous inoculation of T24 cells with hWJMSC or hWJMSC-MVs into male BALB/c nu/nu mice. Tumor incidence in group T24 cell alone (control group) achieved to 100%. In contrast, there were 72.22% (±19.24) and 44.44% (±9.62) T24 xenografts observed in group T24 + hWJMSCs and T24 + hWJMSCs-MVs, respectively. Compared to control group and T24 + hWJMSCs, hWJMSC-MVs significantly decreased the tumor incidence ((44.44±9.62) % versus 100%, P<0.01; (44.44±9.62) % versus (72.22±19.24) %, P<0.01)) (**Figure 8B**). Meanwhile, subcutaneous injection of hWJMSC-MVs alone didn’t form any tumor nodules (data not show). Tumor nodules were first observed at 6 days in group T24 cells alone or mixed with hWJMSCs after injection. However, there were tumor nodules with a diameter of 0.5 cm observed within 9 days after T24 cell injection in the presence of hWJMSC-MVs (**Figure 8A**). This xenograft study showed that bladder tumor T24 cells grow quickly after tumor nodule formation. Tumor volume of the three groups (T24 alone, T24+hWJMSCs, T24+hWJMSC-MVs) achieved to 4463.13±1209.77, 1843.50±768.94 and 174.87±68.88 mm^3^, respectively. There were significant differences within the group and between the groups compared to respective controls (**Figure 8B**). As expected, hWJMSC-MVs, as well as hWJMSCs, significantly reduced the tumor burden as observed by measuring tumor size (**Figure 8D, E**). The weight of the mice had no significant difference between the groups at days 12 and 24 (**Figure 8C**).

### Histopathology and immunohistochemistry

**Figure 9:**
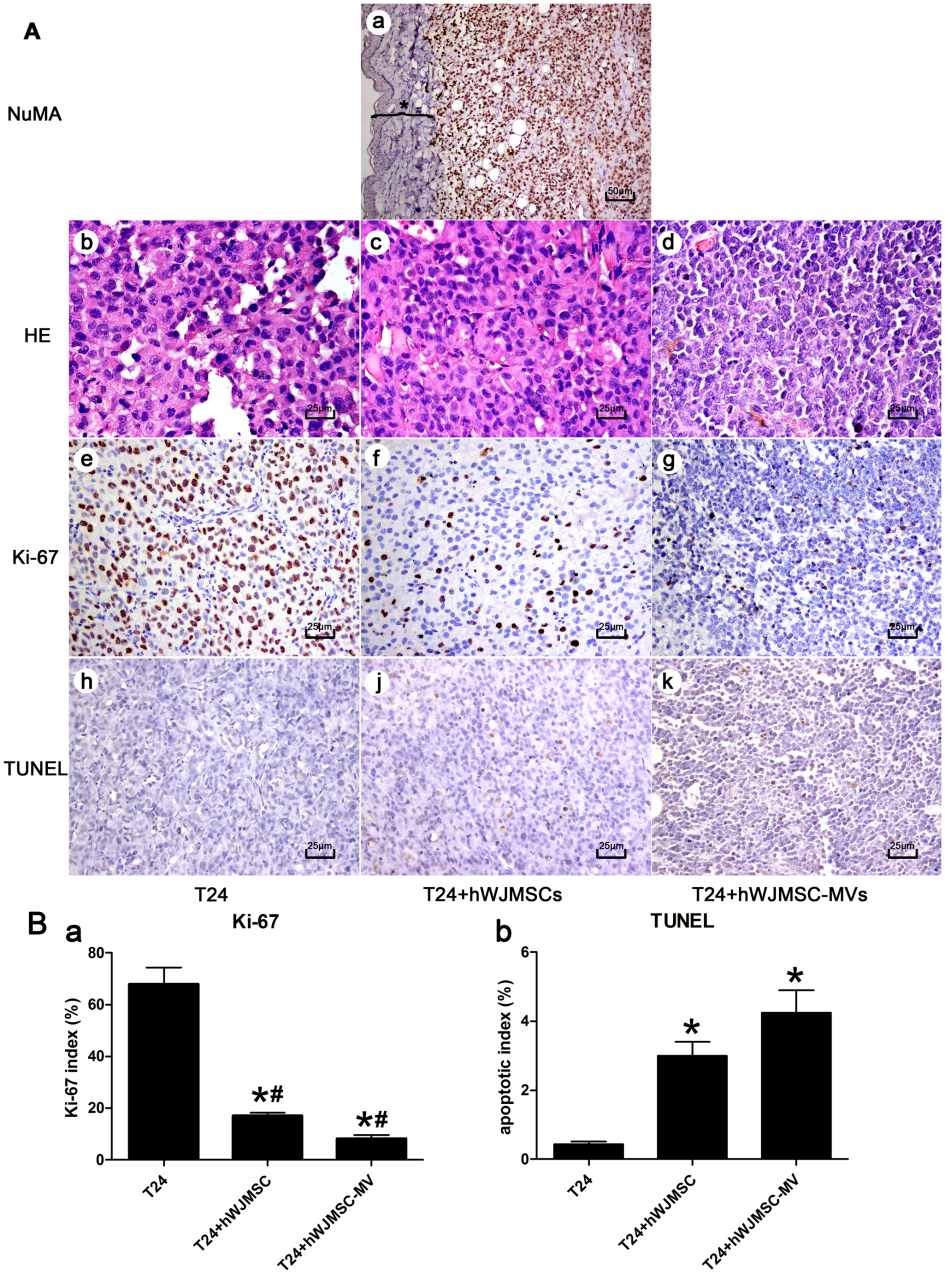
Histopathology and immunohistochemistry of T24 xenografts. A, a: T24 xenograft sections were immunostained by human NuMA. All the solid tumor cells were strongly positive for NuMA, but absent in mouse dermal and subcutaneous tissue cells (black asterisk). A, b–d: Histological analysis was performed by H&E staining after 28 days of T24 cell transplantation: T24 xenografts were composed of malignant irregular cells with hyperchromatic nuclei and active mitotic figures. Compared with the control group (A, b, T24 cells alone), tumor cells exposed to hWJMSCs (A, c) or hWJMSC-MVs (A, d) were found to be smaller in nuclear size and short of extracellular matrices. A, e–g: immunostaining against nuclear antigen Ki-67. hWJMSC-MVs, as well as hWJMSCs, significantly decreased the Ki-67 positive tumor cells in comparison with the control group receiving only T24 cells (P<0.01) (B, a). A, j–k: Apoptosis of T24 xenografts were evaluated by TUNEL assay. Results were expressed as percentage of TUNEL positive cells. Increased apoptosis indices were observed in T24 xenografts of the groups: T24+hWJMSCs, T24+hWJMSC-MVs, compared to the control (T24 cells alone) (B, b). The “*” and “#” indicate statistical significance at P<0.05 compared with respective controls within the group and between the groups, respectively. a, scale bar  =  50 µm; b-k, scale bar  =  25 µm.

Grossly, the T24 cell xenograft possessed an invasive growth pattern, having no definite border and invading into the muscle layer (**Figure 8E**). Multifocal tissue necrotic areas were observed in some bigger tumor body transverse sections, predominantly in the control group (T24 cells alone). Microscopically, immunostaining showed all the solid tumor cells were strongly positive for human NuMA, but absent in mouse dermal and subcutaneous tissue cells (**Figure 9A** **, a**). Histopathologically, T24 xenografts were composed of malignant irregular cells with hyperchromatic nuclei and active mitotic figures (**Figure 9A** **, b, c, d**). Tumor cells in the presence of hWJMSC-MVs (**Figure 9A** **, c**) were smaller in nuclear size and short of extracellular matrices in comparison with group T24 cells alone (**Figure 9A** **, b**). Ki-67 labeling indices were approximately 68% (±6.44), 17.13% (±1.21) and 8.23% (±1.36) in bladder tumor T24 xenografts for groups T24 cells alone (**Figure 9A** **, e**), T24+hWJMSCs (**Figure 9A** **, f**) and T24+hWJMSC-MVs (**Figure 9A** **,** **g**), respectively. Our data analysis found that the Ki-67 index difference between the three groups was greatly significant (P<0.01) (**Figure 9B** **, a**).

### TUNEL assay

Apoptosis of T24 xenograft tissue was assessed by TUNEL technique and was quantified as percentage of TUNEL positive cells. Increased apoptosis indices were detected in T24 cells co-inoculated with hWJMSCs or hWJMSC-MVs into mice compared to only receiving T24 cells ((3.00±0.41) % versus (0.43±0.08) %, P<0.01; (4.25±0.60) % versus (0.43±0.08) %, P<0.01; respectively)) (**Figure 9A** **, j, h, k; 8B, b**). However, no significant difference was observed in the percentage of TUNEL positive cells between groups of T24+hWJMSCs and T24+hWJMSC-MVs.

## Discussion

MSCs can play a dual role in cancer progression by secreting multiple bioactive factors that orchestrated tumor growth [34]–[36]. And stimulatory and inhibitory effects may represent a balance between pro- or anti-survival factors. Emerging evidence suggests that MSCs show antitumor effect upon multiple tumor cells, such as Kaposi’ sarcoma [1], glioma cells [37], breast cancer [3], [4], [38] and Non-Hodgkin’s Lymphoma [2]. Recently, much attention has been given to MVs, which play a vital role in cell-to-cell communication that may re-program target cells through membrane transfer in response to activation signals [24], [26]. MVs transfer not only membrane components but also nucleic acids (functional mRNAs and miRNAs) between different cells, emphasizing their role in the intercellular communication [20], [27], [39], [40]. MVs provide a novel avenue ensuring short- and long-range exchange information between different cell types, which may be a potential mechanism underlying MSCs’ tumor-suppressive properties. Several groups reported no transformation of MSC after long term culture [41], however, other researchers have reported spontaneous transformation of hMSC, which may favor tumor growth [7], [42]. In this regard, MVs administration may have more advantages given the therapeutic potential of MVs. In this study, we successfully isolated MVs from hWJMSCs’ supernatant characterized by heterogeneous lipid bi-layer vesicles ranging from 30–500 nm and expressed CD9, CD44, CD63, CD73. Therefore, understanding the modulation of MVs inhibitory effect upon tumor cells may provide insight into the molecular mechanisms that underlie MSCs antitumor effect which includes anti-proliferative and pro-apoptotic effect in vitro and in vivo.

Our data demonstrated that MVs inhibit tumor cell growth in two experimental systems, including cell culture in vitro and animal transplantation model for tumorigenesis in vivo. And the specificity of hWJMSC-MVs was verified by the lack of anti-tumor effect of MVs derived from human foreskin fibroblasts. The in vitro study found that hWJMSC-MVs abolished tumor cell proliferation via G_0_/G_1_ phase arrest in a dose-dependent fashion. p21 acts as an inhibitor of cell cycle progression, which plays a major role in tumor growth process. The p53 tumor suppressor proteins can upregulate p21 transcription via a p53-responsive element [43]. Upon treatment of hWJMSC-MVs, both p-p53 and p21 were dramatically up-regulated, which may explain why T24 cells were blocked in G_0_/G_1_ phase. Furthermore, we confirmed this point in T24 cell transplantation mouse model. Ki-67 protein is a nuclear antigen tightly associated with somatic cell proliferation [44], [45], which is present during the active cell cycle (G_1b_, S, G_2_ and M phase), but is absent from resting cells (G_0_, G_1a_ phase) [33], [46]. A recent large-scale study stressed the importance of Ki-67 labeling index as a useful biomarker of urothelial carcinoma outcome [47]–[49]. Immunostaining of T24 xenografts have shown that the Ki-67-reactive nuclear antigen appeared in a large number of T24 groups. In contrast, T24 cells seemed to lose the expression of antigen Ki-67 when exposed to hWJMSC-MVs, and were arrested in the G_0_/G_1a_ phase of the cell cycle, which may lead to the sluggish growth of tumor cells. Similar findings were observed by Philip K. Lim et al. that bone marrow stroma cell-derived exosomes containing microRNAs contributed to breast cancer cell quiescence [50]. And Lu-yan Rong et al. also found that MSCs exerted potential inhibitory effects on tumor cell growth by G_0_/G_1_ phase arrest of cell cycle [51].

Besides arresting T24 cells in G_0_/G_1_ phase, hWJMSC-MVs have also shown pro-apoptotic properties both in vivo and in vitro. Similar to Aarif Y. Khakoo’s finding [1], we observed that tumors in animals receiving only T24 cells had more multifocal tissue necrotic areas in comparison with tumors derived from animals co-inoculation with T24 cells and hWJMSC-MVs. Furthermore, we found co-injection of T24 cells with hWJMSC-MVs in mice led to an initial delay in tumor formation despite no significant differences between the groups. Mice subcutaneously injected with T24 cells mixed with hWJMSCs-MVs had a much lower tumor incidence and a smaller tumor volume than the control group. Similar results were observed in mice co-transplanted with T24 and hWJMSCs, but with lower magnitude. To elucidate the mechanism by which hWJMSC-MVs attenuate T24 cells growth, we hypothesized that Akt and Caspase 3 may be involved in this process. We analyzed the Akt/p-Akt and cleaved Caspase 3 by Western blot. Our data revealed that hWJMSC-MVs significantly down-regulated phosphorylation of Akt in a time-dependent manner, whereas there was no significant effect on total expression level of Akt within the groups. Those results suggested hWJMSC-MVs might suppress T24 cell proliferation by inhibiting phosphorylation of Akt. Aarif Y. Khakoo et al. [1] put forward that MSCs induce a contact-dependent inhibition of Akt activation in vitro which result in anti-proliferative effect. In contrast, we found that the cell-to-cell contact is seemingly not necessary for MVs-mediated tumor growth attenuation. And the similar effects of MSC-conditioned medium or MSC extracts had also been described by other researchers [38], [52], [53], which also supports this point. Having demonstrated that the physical presence of hWJMSCs is not required for tumor growth attenuation, it remains possible that the signal input may come from MVs, which may exert direct contact with tumor cells in a similar way to intact hWJMSCs. Valentina Fonsato reported MVs derived from human liver stem cell can inhibit hepatoma growth and stimulate apoptosis by delivering selected miRNAs [54]. Therefore, MSCs can transmit miRNAs to tumor cells not only through gap junctions [50], but also via MVs. Considering the association of miRNAs with tumor suppressive and oncogenic functions [55]–[59], we realize that MVs enriched in miRNAs [20], [26], [27], [39], [40] provide a vital avenue to transfer miRNAs into tumor cells that are involved in tumorigenesis. On the other hand, our data also showed that Caspase 3 was activated by hWJMSC-MVs resulting in high expression of cleaved Caspase 3 in a time-dependent manner. These results were consistent with flow cytometry. Namely, hWJMSC-MVs potently induced T24 cell apoptosis predominately via up-regulating cleaved Caspase 3.

Our results are similar to those described by other researchers [51], [60]. However, some other studies had contradicting results, which suggested that MSCs or MSC-derived exosomes may promote tumor growth [36], [61], [62]. Although many mechanisms have been discussed for these observations, no simple paradigm can account for the conflicting findings. The heterogeneity of MSCs and tissue resource may be the major factors contributing to this discrepancy [35].

Taken together, the present study demonstrates that hWJMSC-MVs exert potently anti-proliferative and pro-apoptotic effects on bladder tumor T24 cells both in vitro and in vivo. The mechanism by which hWJMSC-MVs attenuate growth of T24 cells is mainly by restraining phosphorylation of Akt and up-regulating p-p53/p21 and cleaved Caspase 3. These results clearly indicate that hWJMSC-MVs play a fundamental role in hWJMSC induced growth attenuation of bladder tumor T24 cells. Moreover, hWJMSC-MVs may be a potential tool for cancer therapy as a novel cell-to-cell communication.
